# Distinct respiratory responses of soils to complex organic substrate are governed predominantly by soil architecture and its microbial community

**DOI:** 10.1016/j.soilbio.2016.09.015

**Published:** 2016-12

**Authors:** F.C. Fraser, L.C. Todman, R. Corstanje, L.K. Deeks, J.A. Harris, M. Pawlett, A.P. Whitmore, K. Ritz

**Affiliations:** a School of Water, Energy, and Environment, Cranfield University, Bedford, MK43 0AL, UK; b Rothamsted Research, Harpenden, Hertfordshire, AL5 2JQ, UK; c School of Biosciences, University of Nottingham, Sutton Bonington Campus, Leicestershire, LE12 5RD, UK

**Keywords:** Soil respiration, Microbial community, Soil architecture, Complex substrate, Bayesian belief network

## Abstract

Factors governing the turnover of organic matter (OM) added to soils, including substrate quality, climate, environment and biology, are well known, but their relative importance has been difficult to ascertain due to the interconnected nature of the soil system. This has made their inclusion in mechanistic models of OM turnover or nutrient cycling difficult despite the potential power of these models to unravel complex interactions. Using high temporal-resolution respirometery (6 min measurement intervals), we monitored the respiratory response of 67 soils sampled from across England and Wales over a 5 day period following the addition of a complex organic substrate (green barley powder). Four respiratory response archetypes were observed, characterised by different rates of respiration as well as different time-dependent patterns. We also found that it was possible to predict, with 95% accuracy, which type of respiratory behaviour a soil would exhibit based on certain physical and chemical soil properties combined with the size and phenotypic structure of the microbial community. Bulk density, microbial biomass carbon, water holding capacity and microbial community phenotype were identified as the four most important factors in predicting the soils’ respiratory responses using a Bayesian belief network. These results show that the size and constitution of the microbial community are as important as physico-chemical properties of a soil in governing the respiratory response to OM addition. Such a combination suggests that the 'architecture' of the soil, i.e. the integration of the spatial organisation of the environment and the interactions between the communities living and functioning within the pore networks, is fundamentally important in regulating such processes.

## Highlights

• Four different classes of soil respiratory response to complex substrate addition.
• The responses differ with respect to timing and scale of CO_2_ efflux.
• Class type primarily depends on soil structure and microbial community composition.

## 1 Introduction

The rate of plant-derived organic matter decay in soil has been the subject of research for decades (Waksman et al., 1928, Jenny, 1994). However, the relative predominance of the factors governing this still remain a subject of debate (Dungait et al., 2012). Under aerobic conditions, they are considered to fit broadly into four categories: (i) substrate quality (Ågren and Bosatta, 1987), related to the ability of OM to supply both energy and nutrient elements to the decomposer community, as well as intrinsic attributes that determine the decomposability (e.g. hydrophobicity, or content of enzyme inhibiting/complexing molecules); (ii) climate (Aerts, 1997, Jastrow et al., 2006), given that all reactions are governed by thermodynamic principles, their rates rely on both temperature and moisture and are thus likely to change based on climate patterns; (iii) environment (Manzoni et al., 2012), related to the physicochemical properties of the soil (Colman and Schimel, 2013) as well as potential abiotic SOM decomposition (Kemmitt et al., 2008); (iv) soil biota (Ettema and Wardle, 2002, Dungait et al., 2012), manifest via the constitution and physiology of the microbial community and associated enzyme pools.

Despite the majority of the breakdown and turnover of OM being an inherently biological process, soil microbial communities have been largely neglected as a factor in most modelling frameworks until recently (Allison, 2012, Colman and Schimel, 2013, Wieder et al., 2013). Even empirical studies suggest that biology (mostly in terms of the microbial community composition) plays only a secondary role in determining the rates of these processes which are governed in large part by climate (temperature and rainfall – e.g. Aerts, 1997, Jastrow et al., 2006) and the chemistry of both the soil environment (e.g. Frey et al., 2004) and the SOM being degraded (Reed and Martiny, 2007, Makkonen et al., 2012). Determining the relationship between microbial population size or diversity and OM mineralisation has proved difficult (Fierer et al., 2007), evidenced by the apparent insensitivity of rates of OM mineralisation to changes in abundance (Jenkinson and Powlson, 1976, Garcia-Pausas and Paterson, 2011) and diversity (Garcia-Pausas and Paterson, 2011, Berthrong et al., 2013) of microbial communities. Furthermore, some current theories suggest the high energetic costs of acquiring nutrients and energy from soil OM limits the capacity of the microbial community to degrade humified material (Ekschmitt et al., 2005). Taken together, these ideas have given rise to the Regulatory Gate hypothesis (Kemmitt et al., 2008). This postulates that the disproportionately rapid rate of respiration observed after chloroform fumigation of a soil has killed off the majority of the microbial population is because the mineralisation of soil OM is a two stage process (Kemmitt et al., 2008). The first, and rate-limiting step, is abiotic and independent of microbial processes, followed by the second step where microbes are able to mineralise the small, now biologically-available, substrates. The mode of abiological conversion is not clear but could be chemical oxidation or hydrolysis, desorption from the solid phase, diffusion from pores/aggregates otherwise inaccessible to microbes, the action of extracellular enzymes or, more likely, some combination of these (Kemmitt et al., 2008). This is at odds with much of the literature, which presumes that intact microbial cells are required to turn over the majority of SOM, and where the focus is on what factors are that control the rates at which this happen (Kuzyakov et al., 2009, Paterson, 2009).

Decomposition of OM by microbial communities is highly dependent on the environmental context (extrinsic factors – those that relate to the soil as it occurs in the landscape e.g. land use, slope, and aspect). However, because soil physicochemical properties span such a wide range of variables (intrinsic factors – those which characterise soil at a specific site e.g. texture, SOM, and pH), few studies have been able to include a range of such variables simultaneously. This has led to in-depth understanding of how individual intrinsic factors such as pH (Rousk et al., 2009, Rousk et al., 2011, Whittinghill and Hobbie, 2011) or texture (Wang et al., 2003) influence OM turnover, but offers little insight as to how these factors interact with others as part of a complex network of influences. Similarly, many studies on the effect of litter quality rely on laboratory incubations where different types of litter/substrates are added to a restricted range of soil types (e.g. Gunnarsson et al., 2008, Rinkes et al., 2014), or on reciprocal transplant experiments (Reed and Martiny, 2007) where soil types and physicochemical factors are generally not considered.

Many studies have found that the addition of carbonaceous substrates has an effect on the composition of the microbial community (e.g. Griffiths et al., 1998, Eilers et al., 2010, Pascault et al., 2013, Jagadamma et al., 2014), though only variable effects on function have been observed (Cong et al., 2015, Schimel and Schaeffer, 2012; Veen et al., 2015a, Veen et al., 2015b, Zak et al., 2003). Frequent assertions are made of the so-called ‘home field advantage’ – the increased ability of microbes to decompose litter from their native plant communities (Ayres et al., 2009, Keiser et al., 2014, Strickland et al., 2009, Wallenstein et al., 2013) – alongside almost as many instances where this is sought but not found. When complex substrates, i.e. those comprising a variety of compounds, including most forms of naturally-derived organic materials, are added to soils the subsequent respiratory profile is often complex (Ayres et al., 2006, Gunnarsson et al., 2008, Rinkes et al., 2014). This coupled with the observation that the *response* to added complex substrates differs between soils from different settings (Grayston et al., 2001) suggests that soil-specific OM mineralisation pathways exist, governed by combinations of biotic and abiotic system properties. This leads to questions about whether our understanding of OM cycling can be improved with a greater understanding of the impact of the size and structure of the soil microbial community (Schimel and Schaeffer, 2012) and whether this understanding can ultimately lead to improvements in earth system model performance (e.g. Schimel, 2013, Wieder et al., 2013).

The wide variation in OM quality and quantity, the impact of multiple environmental conditions, and inherent variation in microbial processes, contribute to the significant difficulty in unravelling the importance of microbial communities to OM decomposition, and subsequently how their effect into models of terrestrial C-cycling. The overarching purpose of this work was to assess the short term ability of extant microbial communities, in a diverse range of soils, to process inputs of a plant-derived complex organic substrate. This will aid our understanding of the relationship between microbiota, the combination of intrinsic and extrinsic factors making up the soil environment, and the manner in which OM is mineralized by such communities.

We hypothesized that on addition of a complex organic material (i) distinct respiratory response patterns would be manifest in different soils arising from their variety of intrinsic and extrinsic factors, (ii) such responses would be related to combinations of particular factors rather than being driven by individual factors and, (iii) because CO_2_ release from soils is an intrinsically biological process, the size and structure of the microbial community would be the key factors in determining the respiratory response to additions of OM.

## 2 Methods

### 2.1 Sample collection and preparation

Sites were identified through a random stratified subsample of the 677 locations of the National Soil Inventory (NSI) of England and Wales (McGrath and Loveland, 1992) based on prior information about pH and carbon content of soils. Sites were categorised as high, medium and low pH (low<6.5, medium 6.5–7.5 and high >7.5), then further as high, medium and low carbon contents (low<1.65%, medium 1.65–2.55%, and high >2.55%), resulting in 9 categories of site; final selection for sampling was based on location and permission to access land being granted. Corstanje et al. (2015) found that to successfully determine the relative importance of factors to soil processes, using Bayesian modelling, it was critical to have data on soil properties (both intrinsic and extrinsic) cover as wide a range of values as possible. For this reason within field variability was not considered (samples were pooled as described later) so that samples could be collected from a large number of sites instead. Sixty-seven sites were sampled (see Table S1 for details) by collecting 25 cores (3.5 cm diameter by 10 cm depth) in a grid pattern over a 25 m^2^ area. Three further samples were collected from the centre of the sampling grid at each site and their bulk density determined. Site-specific information about land use, length of land-use, slope and aspect (extrinsic factors) was also collected. The 25 cores were pooled and taken as representative of the site, transported to the laboratory and stored at 4 °C until processing (as was done for the NSI). Pooled samples were sieved to pass a 2 mm mesh screen and a subsample was dried at 105 °C for 48 h to determine moisture content. The water holding capacity was determined using the saturation and drain method modified from Harding and Ross (1964); briefly, a 50 g sample of sieved soil was placed in a funnel, saturated with 100 mL of de-ionised water for 30 min prior to being allowed to drain for a further 30 min. The volume of water drained was combined with the pre-determined moisture content to calculate the effective water holding capacity. This was then used to adjust 200 g of each soil to 45% of their respective water holding capacity. Moisture adjusted soils were then pre-incubated at 25 °C for 7 days to avoid artefacts caused by the disturbance of sampling and sieving.

### 2.2 Respiration measurements

Samples (0.5 g) of soil were weighed out and mixed with 5 mg of freeze dried powdered green barley (*Hordeum vulgare* L.) shoots hereafter denoted ‘substrate’ so as to supply 2.25 mg C g^−1^ dw equivalent soil. The 5 day time-course of CO_2_ evolution at 6-minute intervals immediately following mixing was determined independently for each of 6 laboratory replicates per site using an automated multi-channel conductimetric respirometer (RABIT, Don Whitley, Shipley, UK (Butler et al., 2011);). We used the larger number of laboratory replicates as we were interested in the reproducibility of the response.

### 2.3 Soil properties

A range of soil properties were measured for all sites; pH was determined by shaking 10 mL of soil in 50 mL of deionised water for 30 min, the suspension allowed to settle and measured using a Mettler Toledo MA235 pH meter. Total carbon and nitrogen were determined by elemental analysis (Elementar, Vario EL III). Soil organic carbon was estimated gravimetrically by loss on ignition at 430 °C for 12 h. Percentage sand, silt and clay were measured using the pipette method (Day, 1965) and bulk density was obtained by drying 3 cores of known volume at 105 °C and calculating the dry weight of the volume of soil. Microbial biomass-C (MBC) was determined by chloroform fumigation extraction (Jenkinson and Powlson, 1976) using a K_EC_ of 0.45 (Vance et al., 1987). The phenotypic structure of the microbial community was measured using phospholipid fatty acid analysis (PLFA) (modified from Frostegård et al., 1993) where lipids were extracted from 10 g of freeze dried soil using Bligh and Dyer solvent (1:2:0.8 (v/v/v) chloroform: methanol: citrate buffer). Lipids were then fractionated using solid phase extraction cartridges (ISOLUTE SI (unbonded silica sorbent), Biotage, P/N 460-0050-C) and methylated. The resultant fatty acid methyl esters (FAMES) were analysed by gas chromatography (6890 N Agilent Technologies) using an HP-5 capillary column (30 m length, 0.32 mm ID, 0.25μm film) coated with 5% phenylmethyl siloxane. The temperature programme was as follows: 50 °C (1 min) ramping to 160 °C at 25 °C minute^−1^, followed by increases of 2 °C minute^−1^ to 240 °C and finally 25 °C minute^−1^ up to a maximum of 310 °C for 10 min. The injector temperature was set at 310 °C, the flame ionisation detector at 320 °C, and the He flow set at 1 mL min^−1^. Sample profiles were subsequently determined using G2070 Chemstation for G.C. systems software. FAMES were identified via comparison of retention times to those of a standard bacterial acid methyl ester mix (Supelco Inc.) and the relative abundances (mol %) of indicator fatty acids determined.

### 2.4 Descriptive modelling and parameter extraction

**Fig. 1:**
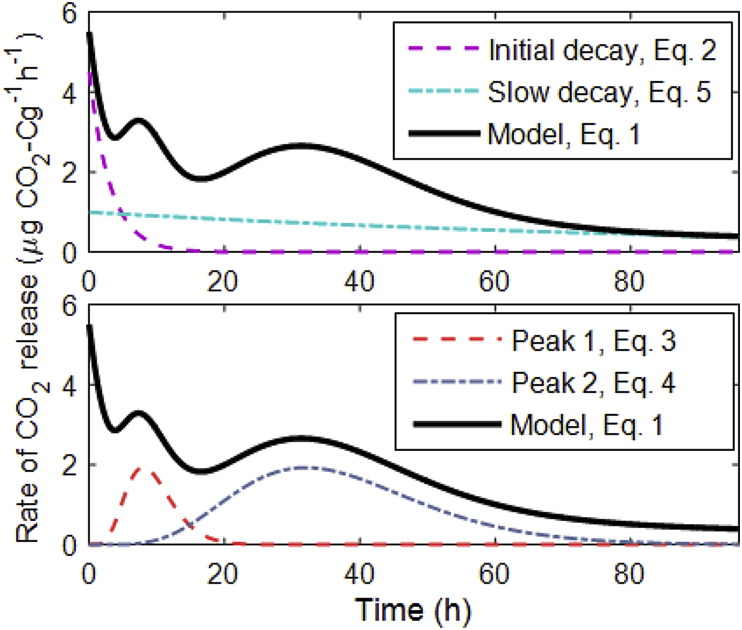
Four components of the descriptive model fitted to the experimental data. Top panel shows the initial decay (purple – Eq. (2)) containing information about *B* – the amplitude of the peak, and *k* – the decay rate alongside the slow decay function (green) which was fixed at 0.1 – Eq. (5). Lower panel shows the components describing the secondary (red – Eq. (3)) and tertiary (blue – Eq. (4)) peaks containing information about the time to peaks (*τ*_*2*_ and *τ*_*3*_), amplitude of peaks (*A*_*2*_ and *A*_*3*_), and the area under each curve (*Area*_*2*_ and *Area*_*3*_). The black line is the same in each panel and shows the summation of all 4 parts to give the descriptive model as a whole (Eq. (1)). (For interpretation of the references to colour in this figure legend, the reader is referred to the web version of this article.)

A descriptive model was fitted to the rate of CO_2_ evolution data in order to quantify the key characteristics of the response profiles to the substrate addition. The model (Eqn. (1)) is a summation of four components (Fig. 1), each describing a different parts of the response that are assumed to relate to the decomposition of different components of the complex substrate that was added to the soil. Thus, the respiration rate, *Y* (μg CO_2_-C g^−1^ h^−1^), is given by

(1) $$Y=y_{1}+y_{2}+y_{3}+y_{4}$$

The first component, the initial decay (*y*_*1*_), is an exponential decay function primarily describing the initial few hours of the data:

(2) $$y_{1}=Be^{-kt}$$

Where *B* (μg CO_2_—C g^−1^hr^−1^) is the amplitude of the peak, *k* (h^−1^) is the decay rate and *t* (h) is time.

The second component (*y*_*2*_) describes the secondary pulse (i.e. increase in respiration and later decline) that was observed in many of the experimental responses:

(3) $$y_{2}=\frac{A_{2}t^{\left(\alpha_{2}-1\right)}e^{-\frac{t}{\theta_{2}}}}{\tau_{2}^{\alpha_{2}}\text{Γ}\left(\alpha_{2}\right)}$$

Where $\theta_{2}=\tau_{2}/\left(\alpha_{2}-1\right)$, *A*_*2*_ (μg CO_2_—C g^−1^hr^−1^) is the amplitude, *τ*_*2*_ (h) is the time to peak, *α*_*2*_ (−) is a shape factor related to the width and the skew of the peak, and Γ is the gamma function.

The third component (*y*_*3*_) represents the tertiary pulse observed in many of the responses is described similarly:

(4) $$y_{3}=\frac{A_{3}t^{\left(\alpha_{3}-1\right)}e^{-\frac{t}{\theta_{3}}}}{\tau_{3}^{\alpha_{3}}\text{Γ}\left(\alpha_{3}\right)}$$

Where $\theta_{3}=\tau_{3}/\left(\alpha_{3}-1\right)$, *A*_*3*_ (μg CO_2_—C g^−1^hr^−1^) is the amplitude, *τ*_*3*_ (h) is the time to peak, and *α*_*3*_ (−) the shape factor relating to this tertiary peak.

The fourth component, the slow decay (*y*_*2*_), is an exponential function describing the tail end of the response curve using a single parameter for the decay rate. As the other model parameters were sensitive to this parameter, and the respiration rate in most of the soils returned in a similar manner towards the end of the period for which measurements were made, the decay parameter was fixed at a value of 0.1, thus:

(5) $$y_{4}=e^{-0.1t}$$

The total rate of respiration was then modelled as a summation of these four components. The model parameters were estimated for each soil by fitting Eqn. (1) to the experimental data for all 6 replicates simultaneously. The model parameters were fitted using the MATLAB (2015b) function lsqcurvefit (default settings) which minimises the least squares of the model fit to the data (MATLAB, 2015b). The imposed model structure assumed that two distinct peaks in the response occurred, corresponding to equations (3), (4)) respectively. As such, the model parameters associated with these equations could only be identified with confidence when two distinct peaks were observed in the data. The variance associated with each parameter estimate was therefore calculated (using the Jacobean matrix outputted by *lsqcurvefit*) and these variances were used to assess when the imposed model structure was appropriate.

### 2.5 Statistical analysis

The PLFA profile data was analysed using principal component analysis (PCA) on the correlation matrix (JMP version 11 (SAS Institute Inc.)), the resultant factor scores were incorporated into the larger data set for use as detailed below.

Analysis was carried out in two stages; first the estimated model parameters were analysed using a minimum variance, hierarchical clustering approach using the Ward method (Ward, 1963) in order to identify when there was consistent behaviour in the respiratory responses. These groupings were confirmed by subjecting the modelled parameter values to PCA using the correlation matrix and visualising the factor scores from the first 3 components (using JMP version 11; SAS Institute Inc.). The second stage of analysis took the clustering obtained and related it to the soil property data by means of a Bayesian belief network (BBN), this was carried out in Netica version 5.15 (Norsys Software Corp.). Bayesian Belief Networks are a graphical representation of a probabilistic dependency model. They represent the variables that affect the response of interest in the form of a graph or network and describe the relationships between the drivers and responses as a set of conditional probabilities (Talaab et al., 2015). Soil property, microbiology, land management, and environmental context data were combined in the BBN and used to predict cluster identity for each site. Initially all variables were included in the model and subsequently non-informative variables (based on variance accounted for) were removed to give the most parsimonious net for predicting cluster. The conditional probabilities produced for each cluster by the BBN were visualised as a heat map using R version 3.2.2 (R Core Team, 2016).

## 3 Results

### 3.1 Soil properties

Across the population of soils sampled, wide ranges of soil properties were observed (Table 1). Sites were subsequently classified into 6 different land management types: pasture (19), arable (26), forestry (7), moorland (4), vegetables (9) and other (2 sites: 1 bog and 1 playing field), across the 67 sites sampled, 46 unique soil associations were observed, these were further amalgamated into 10 representative soil groups (RSG) – full details of properties relating to each site are presented in Table S1.

### 3.2 Model fitting and parameter clustering

**Fig. 2:**
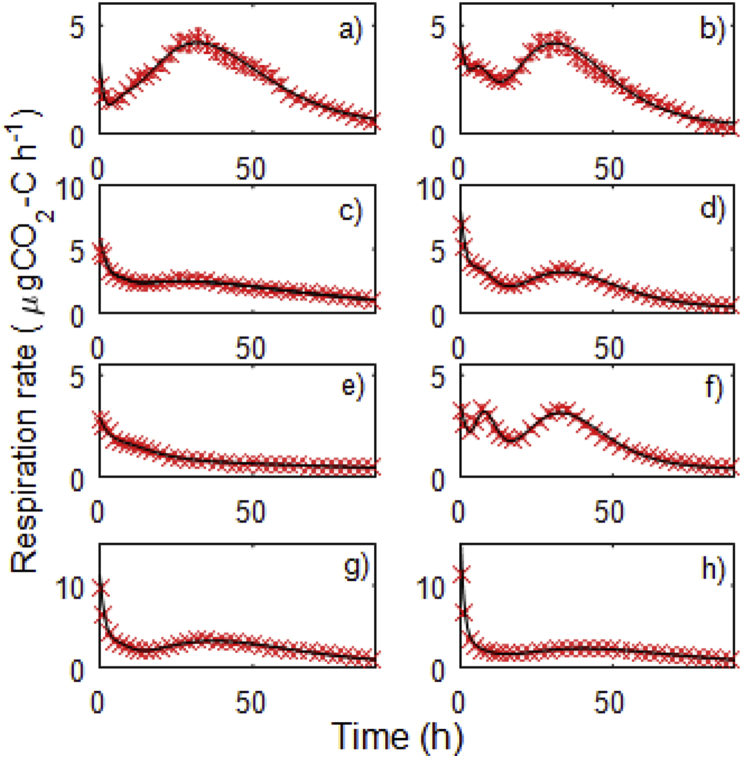
Representative examples of the observed (crosses) and modelled (lines) respiration profiles after the addition of the substrate to diverse soils. The example soils are (a) Soil 33; (b) Soil 57; (c) Soil 66; (d) Soil 17; (e) Soil 63; (f) Soil 23; (g) Soil 56; (h) Soil 52, full details about all soils can be found in Table S1’.

**Fig. 3:**
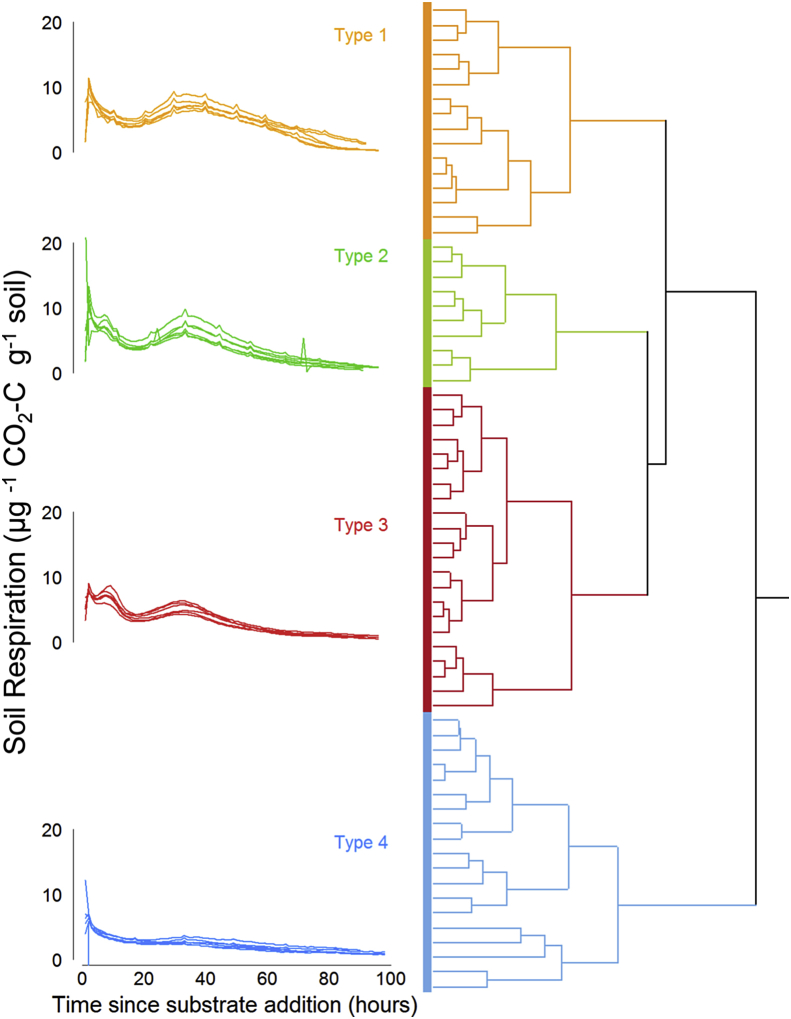
Results of hierarchical clustering of the model parameters listed section 2.5. showing 4 archetypical respiratory responses to the addition of organic matter. Laboratory replicates show a high degree of reproducibility within one site as is seen in the example plot on the left hand side where **each plot is 1 site** and *n* = 6.

A range of respiratory responses were observed over the 5 days following the addition of the substrate and were well captured by the model (Fig. 2). Hierarchical clustering of the fitted model parameters identified 4 clusters in the data, reflecting 4 modes of respiratory behaviour (Fig. 3). ‘Type 1’ was characterised by a large initial flush between 5 and 10 h followed by a sustained pulse of respiration peaking between 35 and 40 h after the beginning of incubation, and this behaviour was observed in approximately 23% of the soils (n = 16). ‘Type 2’ soils also exhibited a large initial flush in the first 5–10 h followed by a secondary pulse between 10 and 20 h and then a tertiary pulse peaking between 35 and 40 h after the beginning of incubation, this type of behaviour was exhibited by 15% of soils observed (n = 10). The most common behaviour, Type 3, was observed in 33% of cases (n = 22), characterised by a smaller initial flush than Types 1 and 2, followed by a pronounced peak between 10 and 20 h and a third large peak between 30 and 40 h after the addition of substrate (Fig. 3). ‘Type 4’ behaviour was seen in 19 soils (28%) and was characterised by an initial flush, usually low, followed by a slow gradual return to a low but stable respiration rate.

**Fig. 4:**
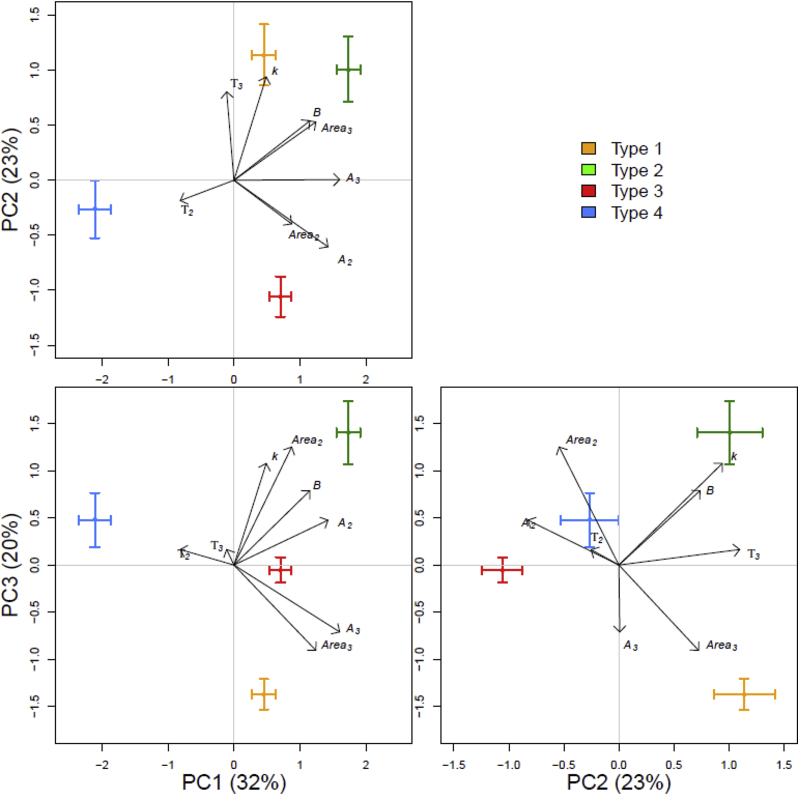
Principal components analysis used to confirm the clustering of sites shown in Fig. 3. The first 3 components account for 75% of the variation; black arrows show the loadings of the model parameters, whiskers = ± 1 SE.

These clustering patterns were confirmed using principal component analysis (Fig. 4; these data are subsequently referred to as the ‘model principal components’). This showed that model PC1 significantly distinguished Type 2 from Type 4 responses, PC2 distinguished Type 1 from Type 3, and PC3 Type 1 from Type 2 (Fig. 4). The loadings of the parameters associated with these PCs were distinct for each type of response (Fig. 4), with Type 1 separation being driven by parameters *k* and *τ*_*3*_ (the decay rate of the initial peak and the time to the third peak), Type 2 distinguished by parameters *B* and *Area*_*3*_ (the amplitude of the first peak and the area under the third peak), Type 3 b y *Area*_*2*_ and *A*_*2*_ (the area and amplitude of the second peak respectively), and Type 4 *τ*_*2*_ (the timing of the secondary peak).

The purpose of fitting the model was to be able to identify statistically different classes of behaviour. As such, it was necessary to fit the same model structure (Eqn. (1)) to all of the responses. After fitting the model the uncertainty in the estimated parameter values was generally small, as indicated by small variance of the parameter estimates (SI Table 2), suggesting that the model structure was appropriate. For some responses though, particularly when the initial respiration rate was low and the secondary or tertiary peaks (e.g. Fig. 2c, e) were not pronounced, parameter identification was difficult. For these responses, the model still described the data well but a simpler model could probably have described the data well also; thus, when fitting Eqn. (1), the variance of the parameter estimates increased (SI Table 2). The variances associated with *τ*_*3*_ (time to peak of the tertiary pulse) were generally larger than those associated with the other parameter estimates. Thus the variance of *τ*_*3*_ was considered representative of the uncertainty in the imposed model structure as a whole. Importantly, 84% of the responses for which the variance of *τ*_*3*_ was greatest occurred in the cluster ‘Type 4’ (i.e. of the 19 soils in which this variance was greatest, 16 soils occurred in Type 4 - note that there are 19 soils in Type 4). Therefore, although a simpler model would have been more appropriate in these cases, the imposed model structure and resulting uncertainty in the parameters did not prevent the cluster analysis from identifying the similarities in these responses.

### 3.3 Relationship between soil properties and respiratory behaviour

**Fig. 5:**
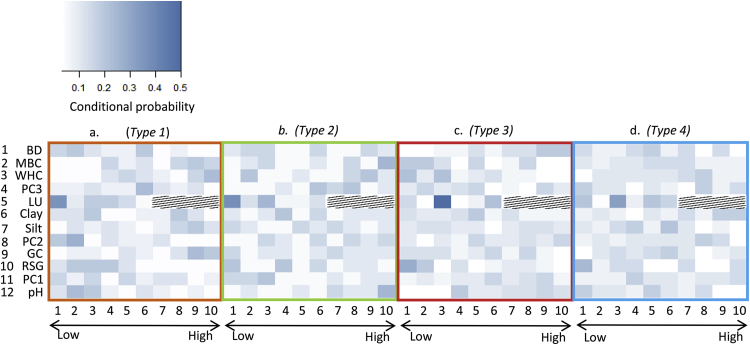
Panels show the results of a Bayesian belief network used to investigate the predictability of respiratory Types from knowledge of soil properties covering physical, chemical, biological, and management aspects of each site (a, b, c, and d show probabilities for Types 1, 2, 3, and 4 respectively). Values of the measured properties were split into 10 bins (where Bin 1 has the lowest values and 10 the highest) as defined for this dataset. For details of bin ranges see Table 2; the more intense the colour the more likely this value is for this type of respiratory behaviour, hatched area indicates empty cells with no value. Soil properties are shown in rank order of importance for predicting Type. BD - bulk density, MBC – microbial biomass carbon, WHC – water holding capacity, PC1, PC2, PC3 – phenotypic principal components, LU – land use, and RSG – representative soil group.

All variables were included to build a naïve BBN (see Table 1 for full list of variables). The sensitivity of respiratory type to the values of the soil properties and land management factors was assessed by the amount that variance in the estimate of Type was reduced by their inclusion in the BBN. This resulted in the retention of 12 variables in the final, parsimonious network (final network shown in Fig. S1), the conditional probabilities of which are shown in Fig. 5 (for ranges of soil properties see Table 2). These probabilities show that sites exhibiting the different types of behaviour had distinct distributions of values for soil properties encompassing biological, chemical, and physical components of the system. The top four soil properties in the hierarchy (shown on the extreme left of Fig. 5), *viz*. bulk density, MBC, water holding capacity and phenotypic PC3 were able to correctly predict respiratory Type 77% of the time. Inclusion of the remaining 8 factors enabled the BBN to correctly classify soils to their observed respiratory type with a 98% success rate (i.e. all but 1 of the 67 soils). Type 1 sites had large conditional probabilities for low bulk density, high MBC, and high water holding capacity as well as mid-range loadings on phenotypic PC3. This contrasted strongly with Type 3 where bulk densities were likely to be greater, MBCs and water holding capacities lower and the loadings on PC3 smaller. The distributions of values of these properties was less well defined for Types 2 and 4 but generally Type 2 sites had low bulk densities, greater MBC and water holding capacities, as well as medium – large loadings on phenotypic PC3. However, there were examples of Type 2 soils with smaller MBCs and water holding capacities. Type 4 sites generally had wide distributions of each of these 4 soil properties.

## 4 Discussion

The addition of a prescribed complex organic substrate elicited 4 distinct types of respiratory response from a large set of diverse soils, in agreement with our first hypothesis. Subsequent analysis of parameters extracted from a descriptive model of the respiration profiles showed that the differences in these parameters could be correlated to both microbiological and physicochemical properties of the soils from each site. It was possible to predict which type of behaviour a soil would exhibit based on prior knowledge of these properties with a 95% success rate (Fig. 5); this was in accordance with our second hypothesis. There was also some evidence in support of our third hypothesis – that respiratory response would be strongly associated with the microbiological properties of the soils. The size of the microbial community (MBC) was identified as one of the most important factors in predicting the respiratory response grouping, the phenotypic principal components derived from the PLFA data also made important contributions to determining these groups.

The differences between the shapes of respiratory response in the 4 different types are i) the magnitude of the respiration rate immediately after substrate addition in Types 1 and 2 compared to Type 3, ii) the number of obvious peaks in respiration rate between Type 1 (2 peaks) and Type 2 (3 peaks), and iii) the very much lower total respiration observed in Type 4 compared to all others. The difference in the size of the initial respiration rate is likely to be linked to the difference in the size of the microbial biomass with Types 1 and 2 having essentially congruent mean values (962 and 960 mg C g^−1^) – compared to the lower average values for Types 3 and 4 (345 and 476 mg C g^−1^ respectively), such that the larger the microbial biomass, the larger the initial flux of CO_2_. The relationship between the size of the microbial community and the size/shape of the CO_2_ respiration rate is intuitive, but apparently complex. This is shown by the divergence in shape of Types 1 and 2 after the initial flush of respiration despite their near identical community sizes and the fact that Type 4 soils had, on average, higher MBC values than Type 3 – corresponding to higher initial release rates of CO_2_ – but over the course of the incubation they had lower total respiration (117 and 183 μg CO_2_—C for Types 3 and 4 respectively). As a result, it appears that the size of the microbial biomass is the primary driver of the maximal rate of respiration occurring in the first 10 h but that the subsequent behaviour is mediated by other, soil-specific, factors, including the phenotypic structure of the community itself. This suggests that recent inclusions of microbiology in earth system models (e.g. Allison, 2012, Wieder et al., 2013) is a positive step but that a detailed understanding of how the biology interacts with the rest of the system is required although differences in scale are highly likely to complicate this picture.

This work suggests that microbes employing this opportunistic strategy exist in all the soils studied (all soils exhibited an initial respiratory flush to a greater or lesser extent during the first 10 h of incubation) although their prevalence or effectiveness differed depending on the environmental context from which they were sampled (Jonsson and Wardle, 2008). For example, Types 1 and 2 have similar mean values of modelled parameter B which reflects the size of this initial respiratory flush and for phenotypic PC1 (−0.711 (±0.128) and −0.780 (±0.143) respectively) but differing mean values for several soil properties 0.7 and 0.8 g cm^3^ bulk density, 0.8 and 0.7 mL g^−1^ WHC, and pH 5.8 and 7.6 for Types 1 and 2 respectively. This highlights potentially high levels of functional redundancy where communities which have marginal phenotypic differences, but originate from different environmental contexts, exhibited this similar initial (first 10 h) respiratory response. Different architectures affect the diffusion of enzymes and substrates or movement of organisms in soil. Therefore, the initial decomposition of added substrate may be subject to a non-biological, rate-limiting step before the community structure has any influence on respiration, as in the regulatory-gate hypothesis referred to previously. Our work differs from what might be expected given that it does not identify SOM (here measured by LOI) as being important to the respiratory response of the soil on the addition of substrate (Colman and Schimel, 2013, Hopkins et al., 2014, Schimel and Weintraub, 2003), though perhaps a consideration of SOM quality would yield different results. This is likely because SOM is closely correlated with, and therefore shares explanatory power with, factors like MBC, bulk density, and texture, all of which are considered highly important by the BBN (this highlights the need for careful interpretation of parameters that are not entirely independent in these complex systems). Also, given that the respiratory response here is a substrate induced one, the most important part of the SOM is likely to be the active portion represented by the MBC. pH is often reported to have a strong relationship to respiration (e.g. Rousk et al., 2009, Rousk et al., 2011) and microbial phenotypic community structure (Creamer et al., 2015). Interestingly, whilst pH did feature in our final BBN, it was the least important of the factors included. However, the hierarchical position of these factors in the ranking suggests that microbial community structure is the more important and that the influence of pH is more subtle, this possibly reflects the inclusion of land use in our analysis which is likely to share much of the predictive power of pH.

The substrate used here was a green plant material whereas many previous studies have used senesced plant material (e.g. leaves or straw) which represents a qualitatively different kind of substrate (Rinkes et al., 2014, Gunnarsson et al., 2008). Senesced materials will include fewer sugars, amino sugars and proteins than green material, thus increased fragmentation by extracellular enzymes will be required to transform these into microbially assimilable forms. It is known that the majority of C mineralized in the first 24 h after substrate addition is derived from free sugars, fructans and soluble organic-N compounds (Gunnarsson et al., 2008) and that plant residues with high concentrations of these compounds have higher initial rates of respiration than residues with lower concentrations when added to samples of the same soil under the same circumstances. Very low rates of respiration in the Type 4 soils, despite their generally higher SOM and total C contents (although this is variable across the group of soils), suggest the absence of a priming response in these 19, however as this is not an isotope tracer study, it is impossible to tell the origin of the CO_2_—C and therefore an assessment of priming is difficult.

## 5 Conclusion

Soil respiration after complex substrate addition is the product of a set of diverse interacting factors within the system rather than being dominated by individual driving factors, separating clearly into four distinct Types. This argues for the inclusion of soil microbial factors – both in terms of population size and community structure – in models of soil C-cycling alongside more traditionally modelled variables such as pH, temperature, and moisture. It is notable that the four soil properties which ranked highest in the BBN (bulk density, MBC, WHC and phenotypic PC3) can be considered as relating to the inherent physical and biological structures of the soil and the microbiology therein. Bulk density will be related to the porosity of the soil, and the WHC a surrogate for the nature of the pore network, particularly with respect to the delivery of substrate and oxygen to microbes via diffusion. MBC and phenotypic PC3 relate to the size and phenotypic constitution of the microbial communities (noting that phenotypic PCs 1 and 2 also feature within the dominant properties arising from the BBN). This combination of structural and biotic factors could be construed as the ‘architecture’ of the soils, a term which integrates the spatial organisation of the environment and the interactions between the communities living and functioning within the pore networks (Ritz and Young, 2011). This makes the case that such architecture is fundamentally important in governing the decomposition of organic substrates entering soils. We posit that it suggests respiratory responses may be organised around “attractors” of system type, offering the possibility to further explore state and transition models in soil microbial ecology, which is of great current interest in mainstream ecological research into tipping points, reliability and resilience (Bestelmeyer et al., 2011).’

## Figures and Tables

**Table 1:** Summary statistics for measured soil properties.

Soil properties	Minimum	Median (25th:75th quantiles)	Maximum
Sand (%)	7.5	40 (26.25: 55.15)	88.3
Silt (%)	3.2	30.4 (23.9: 44.95)	69
Clay (%)	0.3	21.6 (16.35: 29.95)	58.7
C:N	5.39	9.34 (8.1: 11.2)	33.41
Total N (%)	0.17	0.3 (0.25: 0.49)	1.81
Total C (%)	1.39	3.08 (2.2: 5.8)	24.62
pH	4.2	6.6 (5.7: 7.3)	8.7
WHC (mL g^−1^)	0.04	0.63 (0.49: 0.81)	1.18
LOI (g g^−1^)	0.027	0.072 (0.047: 0.123)	0.617
MBC (μg g^−1^)	68	397 (284: 778)	2329
Bulk density (g cm^−3^)	0.18	0.98 (0.67: 1.20)	1.53
Slope (°)	0	1.6 (0.5: 4.2)	11
Canopy cover (%)	0	70 (20: 80)	100
Ground cover (%)	0	45 (20: 80)	100
Phenotypic PC1	−7.13	0.64 (0.00: 1.26)	3.52
Phenotypic PC2	−3.50	−0.43 (−1.74: 1.21)	6.90
Phenotypic PC3	−7.95	0.21 (−1.18: 1.23)	5.16

**Table 2:** The ranges or values of soil properties associated with each of the 10 bins used in the BBN. Bins 1–10 correspond to the positions of the boxes indicated in Fig. 5, NA's are shown by hatched areas in Fig. 5.

Soil property	Rank importance	Bin 1	Bin 2	Bin 3	Bin 4	Bin 5	Bin 6	Bin 7	Bin 8	Bin 9	Bin 10
Bulk density (g cm^−3^)	1	0.10–0.49	0.40–0.60	0.61–0.79	0.80–0.89	0.90–0.97	0.98–1.09	1.10–1.17	1.18–1.21	1.22–1.29	1.30–1.54
WHC (mL g^−1^)	2	0.36–0.42	0.43–0.47	0.48–0.51	0.52–0.56	0.57–0.63	0.64–0.70	0.71–0.77	0.78–0.83	0.84–0.97	0.98–1.19
MBC (μg g^−1^)	3	68–169	170–259	260–299	300–359	360–399	400–489	490–599	600–899	900–1499	1500–2400
Phenotypic PC3	4	−7.8–−2.0	−1.9–−1.1	−1.0 –−0.17	−0.16–−0.1	0	0.1–0.22	0.23–0.54	0.55–1.0	1.1–1.6	1.7–6.2
Land use	5	Pasture	Other	Arable	Moorland	Forestry	Vegetables	*NA*	*NA*	*NA*	*NA*
Clay (%)	6	0–11.9	12–14.9	15–16.9	17–19.8	19.9–21.5	21.6–23.9	24–28.9	29–33.9	34–41.9	42–59
Silt (%)	7	3–12.9	13–21.9	22–25.9	26–28	28.1–30.3	30.4–30.9	35–41.9	42–46.9	47–55.9	56–59
Phenotypic PC2	8	−8.6–−2.4	−2.3–−1.6	−1.5–−0.6	−0.5–−0.1	0	0.1–0.39	0.4–0.59	0.6–0.39	1.4–2.29	2.3–6.0
Ground cover (%)	9	0–9	10–19	20	21–30	30	31–69	70–79	80	81–99	100
RSG	10	Stagnosol	Gleysol	Podzol	Cambisol	Umbrisol	Leptosol	Luvisol	Arenosol	Planosol	Histosol
Phenotypic PC1	11	−3.5–−2.6	−2.5–−2.2	−2.1–−1.8	−1.7–−0.10	−0.9–−0.1	0	0.1–0.59	0.6–1.5	1.6–3.5	3.6–6.8
pH	12	4.2–4.6	4.7–5.5	5.6–5.9	6.0–6.3	6.4–6.6	6.7–6.8	6.9–7	7.1–8	8.1–8.4	8.5–8.7

## Appendix A Supplementary data

Table S1 Measured characteristics of 67 sample sites, RSG – representative soil group, *NA* – not applicable. Table S1

Table S2 Model parameters and associated uncertainty measures for each sample site. Table S2

Table S3 Loadings extracted from the phenotypic principal components analysis. Table S3

Fig. S1 Final parsimonious Bayesian belief network diagram; the arrows show the structure of the model with all 12 predictor variables being directly related to respiratory Type. The bars show the distribution of predictor variables across all 67 sites, the range of each parameter value is divided into 10 bins the limits of which are shown to the left of the left hand column with the percentage of sites falling in each bin shown to the right of the left hand column. MBC – microbial biomass carbon, WHC – water holding capacity, RSG – representative soil group, in land use node numbers relate to categories of land use as listed in Table 2; 1 – pasture, 2 – other, 3 – arable, 4 – moorland, 5 – forestry, and 6 – vegetables. Fig. S1
