# Supplementary material for: Distinct respiratory responses of soils to complex organic substrate are governed predominantly by soil architecture and its microbial community
Source: Soil Biol Biochem. 2016 Dec;103:493–501. doi: 10.1016/j.soilbio.2016.09.015 (PMC5113515; doi:10.1016/j.soilbio.2016.09.015)
Supplement: Table S1 — Measured characteristics of 67 sample sites, RSG – representative soil group, NA – not applicable. [file mmc1.pdf]

Soil	Type	Latitude:longitude	sand : silt : clay (%)	RSG*	Soil association	C:N (Total N ; Total C (%))	pH	WHC (mL g^{-1})	LOI (g g^{-1})	MBC (μg g^{-1})	Bulk density (g cm^3)	aspect	slope (°)	Landuse	canopy ; ground cover (%)	Phenotypic PC1	Phenotypic PC2	Phenotypic PC3
1	1	52.4245:-4.0652	7.5 : 53.9 : 38.6	stagnosol	cegin 713d	10.80 (0.70 ; 7.58)	5.5	0.98	0.15	2329	0.54	NA	0	pasture	75; 95	1.403	-2.863	-1.129
2	1	52.4031:-4.0597	26.8 : 45.3 : 27.9	gleysol	conway 811b	10.14 (0.36 ; 3.61)	5.6	0.84	0.07	1226	0.85	NA	0	playing field	60; 85	1.120	-2.485	-1.998
3	1	52.3650:-3.8308	14.6 :69.0 : 16.4	podzol	hafren 654a	10.11 (0.60 ; 6.03)	5.5	0.91	0.15	1303	0.59	SE	8	pasture	80; 100	0.640	-2.715	-2.074
7	1	53.2222:-4.0132	28.8 : 41.8 : 29.4	umbrisol	manod 611c	11.38 (0.83 ; 9.47)	5.1	1.02	0.17	1699	0.51	NE	5	pasture	60; 80	0.510	-3.495	-1.663
8	1	53.2178:-4.0120	25.7 : 43.3 : 31.0	podzol	hafren 654a	13.36 (0.70 ; 9.35)	4.7	1.00	0.17	1717	0.53	N	8	moorland	95; 100	0.623	-1.790	-1.273
19	1	53.2748:-0.7870	36.2 : 28.6 : 35.2	gleysol	fladbury 2 813c	8.30 (0.37 ; 3.08)	6.3	0.56	0.09	435	0.97	NA	0	arable	0; 15	-0.445	0.310	0.209
22	1	52.4699:-1.2493	44.3 : 26.2 : 29.5	stagnosol	beccles 3 711t	9.26 (0.25 ; 2.34)	7.0	0.58	0.07	323	1.08	SW	2	arable	1; 20	1.643	0.210	-0.333
24	1	54.0665:-0.8110	70.1 : 13.1 : 16.8	cambisol	rivington 1 541f	8.31 (0.27 ; 2.27)	5.6	0.57	0.05	341	1.05	N	1	forestry	80; 30	-7.134	0.994	-0.641
26	1	53.0412:-4.0445	34.8 : 48.9 : 16.3	gleysol	conway 811b	10.76 (0.57 ; 6.10)	5.8	0.91	0.13	1407	0.65	SE	1.5	pasture	70; 80	0.799	-1.132	-1.846
27	1	53.0436:-4.0458	32.9 : 55.8 : 11.3	umbrisol	malvern 611a	16.26 (0.82 ; 13.30)	8.1	1.12	0.25	1123	0.33	S	6	forestry	80; 95	0.934	0.162	-1.879
28	1	53.0473:-4.0600	25.6 : 52.0 : 22.4	gleysol	laployd 871a	13.26 (1.22 ; 16.15)	4.2	1.19	0.30	792	0.36	W	10	moorland	50; 100	1.309	0.354	-2.441
33	1	53.4758:-0.6874	43.4: 26.9 : 29.7	podzol	crannymoor 631f	6.18 (0.24 ; 1.50)	4.6	0.52	0.07	339	0.42	E	1.5	moorland	95; 100	-0.979	-0.440	0.804
35	1	52.9988:-4.4290	75.3 : 24.4 : 0.3	podzol	gelligaer 654c	10.75 (0.31 ; 3.37)	5.6	0.70	0.07	739	1.00	SW	6	pasture	90; 85	0.746	-1.220	-1.281
45	1	54.5140:-3.6306	68.5 : 18.0 : 13.6	cambisol	eardiston 1 541c	7.42 (0.29 ; 2.14)	6.7	0.65	0.04	438	1.20	NE	5.5	pasture	90; 30	-3.598	2.228	-7.947
55	1	50.2558:-3.7407	40.0 : 44.1 : 15.9	cambisol	denbigh 1 541j	7.85 (0.49 ; 3.82)	5.9	0.75	0.09	765	0.77	N	10	pasture	100; 90	0.997	-2.069	-0.430
57	1	51.1141:-2.3300	19.3 : 68.1 : 12.6	cambisol	bearsted 2 541B	8.65 (0.28 ; 2.43)	6.7	0.57	0.06	397	1.05	SE	2.5	arable	50; 45	1.193	-2.179	1.071
4	2	50.7769:-3.6021	12.7 : 46.8 : 40.5	cambisol	halstow 421b	8.58 (0.40 ; 3.40)	7.0	0.84	0.14	1545	0.58	NA	0	pasture	80; 60	0.646	-1.809	0.344
12	2	53.1356:-0.4170	73.9 : 10.5 : 15.6	leptosol	elmton 1 343a	6.78 (0.21 ; 1.39)	8.7	0.43	0.04	207	1.39	NW	1.5	arable	45; 25	0.177	-1.220	-0.150
20	2	52.1505:-0.7444	28.0 : 28.1 : 43.8	stagnosol	ragdale 712g	8.67 (0.39 ; 3.41)	8.1	0.78	0.11	440	1.00	NE	3	arable	5; 1	0.471	-1.472	1.865
21	2	53.1822:-0.4890	77.8 : 13.9 : 8.3	planosol	wickham 2 711f	6.83 (0.30 ; 2.07)	8.7	0.46	0.06	279	1.22	E	1.25	arable	20; 100	-0.067	-1.901	1.474
34	2	53.4292:-0.4181	66.0 : 10.3 : 23.7	stagnosol	beccles 1 711r	9.29 (0.39 ; 3.65)	7.8	0.99	0.09	888	0.71	S	0.75	pasture	85; 75	0.468	-2.933	-0.207
41	2	55.4823:-1.7487	49.2 : 31.4 : 19.5	stagnosol	dunkeswick 711p	10.31 (1.28 ; 13.21)	5.0	0.98	0.28	1612	0.19	NNE	3.7	bog	80; 95	1.105	5.028	2.264
52	2	54.1299:-0.2363	33.2 : 36.3 : 30.5	cambisol	panholes 511c	9.49 (0.78 ; 7.38)	7.1	0.87	0.15	1728	0.56	SE	2.5	pasture	100; 80	1.250	3.208	5.160
53	2	52.9541:-4.4811	42.4 : 38.1 : 19.5	arenosol	newport 1 551d	10.34 (0.70 ; 7.27)	6.3	0.91	0.14	1783	0.70	E	5	pasture	60; 75	0.401	-2.672	-0.435
56	2	51.1683:-2.0584	15.7 : 61.0 : 23.3	leptosol	upton 1 342a	33.41 (0.37 ; 12.31)	8.5	0.62	0.07	825	1.06	E	4	pasture	30; 20	1.609	-0.866	2.484
65	2	52.7189:-0.3535	49.9 : 30.4 : 19.8	cambisol	badsey 2 511i	9.28 (0.24 ; 2.22)	8.6	0.47	0.04	294	1.26	SW	0.5	vegetables	0; 5	0.007	-2.182	0.466
9	3	54.4020:-0.5505	52.5 : 30.2 : 17.3	stagnosol	dunkeswick 711p	8.42 (0.54 ; 4.56)	6.2	0.75	0.09	618	0.65	NW	5	forestry	80; 95	-6.360	-0.912	-1.367
10	3	52.9868:0.0599	14.9 : 55.6 : 29.6	gleysol	wisbech 812b	7.72 (0.20 ; 1.57)	8.5	0.57	0.04	155	1.30	NA	0	vegetables	30; 80	1.090	0.319	1.637
11	3	52.9901:0.0579	15.3: 65.6 : 19.2	gleysol	tanvats 811e	8.32 (0.17 ; 1.45)	8.1	0.48	0.03	135	1.20	NW	0.5	vegetables	0; 0	1.380	0.973	2.473
13	3	53.2750:-0.8617	21.3 : 51.7 : 27.0	luvisol	worcester 431	8.19 (0.25 ; 2.07)	6.1	0.61	0.04	288	1.24	W	1.75	arable	10; 80	1.709	-1.722	0.947
14	3	53.1833:-1.1658	80.8 : 13.6 : 5.6	arenosol	cuckney 551b	14.93 (0.37 ; 5.53)	8.1	0.39	0.03	224	1.10	W	2	arable	0; 15	0.500	-1.184	-0.880
15	3	53.2624:-0.3391	51.0 : 27.7 : 21.4	stagnosol	beccles 1 711r	7.84 (0.27 ; 2.15)	7.8	0.40	0.05	255	1.22	NE	1.25	arable	20; 70	0.711	-1.018	0.774
16	3	53.1875:-1.0886	65.5 : 15.2 : 19.3	arenosol	cuckney 551b	10.18 (0.25 ; 2.53)	6.8	0.37	0.03	145	1.53	S	0.75	arable	2; 25	1.013	-0.114	-3.151
18	3	53.1641:-1.2682	39.9 : 38.8 : 21.2	cambisol	aberford 511a	16.71 (0.22 ; 3.61)	8.5	0.45	0.04	299	0.94	E	3.75	arable	2; 40	0.279	-2.155	0.887
17	3	53.3173:-1.1651	35.9 : 50.6 : 13.5	cambisol	aberford 511a	14.72 (0.20 ; 2.96)	6.8	0.48	0.08	385	0.90	NA	0	arable	0; 2	0.356	6.820	1.936
23	3	53.1289:0.0310	50.2 : 25.5 : 24.3	stagnosol	salop 711m	6.64 (0.22 ; 1.45)	7.0	0.48	0.06	266	1.18	SW	0.75	pasture	0;1	2.197	1.459	-2.930
31	3	52.9333:-0.0655	11.1 : 64.7 : 24.2	gleysol	tanvats 811e	8.57 (0.21 ; 1.83)	8.4	0.48	0.04	259	1.10	NA	0	vegetables	75; 60	1.187	1.313	0.974
36	3	54.2692:-1.6015	52.6 : 24.1 : 23.3	cambisol	east keswick 1 541x	6.18 (0.29 ; 1.81)	8.0	0.47	0.04	381	1.42	E	1.12	arable	80; 40	-6.508	0.183	1.210
37	3	54.5836:-1.5966	43.0 : 35.4 : 21.6	stagnosol	dunkeswick 711p	10.72 (0.22 ; 2.37)	6.2	0.57	0.05	395	1.35	SSE	4.2	arable	70; 80	-0.191	1.103	1.748
42	3	54.6725:-2.6063	61.3 : 24.5 : 14.2	stagnosol	clifton 711n	11.05 (0.28 ; 3.08)	6.4	0.67	0.06	658	1.13	NE	1	arable	20; 15	-0.157	-0.430	0.154
43	3	54.6717:-2.6046	33.5 : 46.3 : 20.2	stagnosol	clifton 711n	10.74 (0.25 ; 2.71)	6.3	0.68	0.06	582	1.30	NE	2	pasture	90; 30	0.034	3.756	2.094
46	3	53.8583:-0.6942	79.1 : 5.6 : 15.3	gleysol	everingham 821a	6.40 (0.24 ; 1.55)	6.9	0.41	0.03	201	1.31	S	1.5	vegetables	80; 20	-6.428	1.924	0.802
47	3	54.1229:-0.3029	55.0 : 28.1 : 16.9	leptosol	andover 1 343h	8.66 (0.25 ; 2.19)	7.1	0.50	0.04	379	1.19	NE	5	arable	100; 30	-0.965	2.651	2.310
48	3	53.8545:-0.9213	82.9 : 12.5 : 4.6	gleysol	everingham 821a	11.99 (0.17 ; 2.08)	6.8	0.39	0.04	169	1.23	NA	0	arable	98; 20	0.580	1.401	3.348
49	3	54.0828:-0.7633	52.3 : 27.5 : 20.2	stagnosol	denchworth 712b	5.39 (0.26 ; 1.43)	8.4	0.50	0.03	251	1.21	N	1	arable	80l 20	-5.707	-1.754	-0.001
58	3	50.8520:-2.5547	12.2 : 45.8 : 42.0	planosol	wickham 2 711f	8.29 (0.40 ; 3.35)	6.5	0.70	0.09	631	0.95	NW	1.75	arable	10; 60	0.961	-1.913	0.510
59	3	50.8504:-2.5533	13.3 : 44.6 : 42.1	stagnosol	denchworth 712b	8.42 (0.37 ; 3.09)	6.2	0.78	0.09	797	0.94	W	3.5	pasture	60; 80	2.409	1.496	-0.922
64	3	52.8192:0.6172	74.5 : 13.7 : 11.8	luvisol	barrow 581f	8.39 (0.29 ; 2.41)	8.6	0.41	0.04	120	1.30	SE	5	vegetables	2; 10	0.096	-2.314	0.512
5	4	50.7685:-3.6044	62.7 : 23.3 : 14.0	cambisol	crediton 541e	7.25 (0.25 ; 1.80)	7.0	0.52	0.05	332	1.28	NW	11	arable	20; 10	1.276	-0.626	0.092
6	4	50.7584:-3.6103	19.0 : 55.2 : 25.9	cambisol	halstow 421b	7.15 (0.38 ; 2.68)	6.4	0.64	0.07	492	1.23	W	9	arable	80; 65	1.575	-1.700	1.707
25	4	54.4023:-0.5597	32.9 : 31.5 : 35.6	stagnosol	dunkeswick 711p	11.32 (0.48 ; 5.56)	4.9	0.77	0.11	488	0.34	N	2	forestry	40; 70	-0.864	-0.600	1.246
29	4	50.6944:-3.4309	41.8 : 3.2 : 55.0	luvisol	whimple 3 572f	8.10 (0.30 ; 2.47)	6.7	0.56	0.06	359	1.11	NNW	2.25	arable	70; 30	1.591	2.320	-1.705
30	4	50.6954:-3.4326	40.6 : 39.0 : 20.4	luvisol	whimple 3 572f	9.34 (0.26 ; 2.45)	6.6	0.52	0.06	364	1.16	NNE	4	arable	80; 30	1.800	-0.221	0.002
32	4	53.4757:-0.6901	38.6 : 31.2 : 30.2	podzol	crannymoor 631f	7.39 (0.28 ; 2.09)	4.5	0.48	0.06	292	0.28	N	1	moorland	100; 90	0.076	-2.158	1.732
38	4	54.8984:-1.7516	51.9 : 28.1 : 20.0	stagnosol	brickfield 3 713g	14.96 (0.31 ; 4.69)	6.4	0.73	0.08	631	0.98	SE	6	pasture	70; 60	0.035	3.163	-0.319
39	4	55.3479:-1.6703	39.1 : 33.3 : 27.7	stagnosol	brickfield 3 713g	10.21 (0.29 ; 2.99)	4.5	0.73	0.06	586	0.95	NW	7	pasture	95; 100	0.426	-0.979	1.914
40	4	55.4829:-1.7491	55.3 : 22.4 : 22.3	stagnosol	dunkeswick 711p	10.58 (0.47 ; 4.95)	5.7	0.95	0.12	1543	0.84	NE		pasture	95; 100	-1.208	0.851	1.280
44	4	54.8407:-3.3139	55.7 : 30.6 : 13.7	stagnosol	clifton 711n	7.76 (0.55 ; 4.25)	5.3	0.81	0.08	1071	0.90	NNW	4	pasture	100; 70	-6.180	0.455	-1.232
50	4	53.8063:-0.1641	38.5 : 27.0 : 34.5	stagnosol	holderness 711u	6.97 (0.36 ; 2.53)	6.6	0.62	0.06	375	1.11	NA	0	arable	70; 20	-6.434	0.634	0.644
51	4	53.8973:-0.2362	70.5 : 13.1 : 16.4	cambisol	landbeach 512b	9.95 (0.21 ; 2.06)	6.0	0.45	0.05	276	1.22	SSE	1	arable	5; 40	0.001	0.064	1.019
54	4	52.5822:-0.1382	29.9 : 33.2 : 36.9	gleysol	ireton 873	12.87 (1.00 ; 12.91)	7.4	0.67	0.62	68	0.84	NA	0	vegetables	0; 15	1.759	-0.804	0.618
60	4	52.5258:-0.1796	14.8 : 29.6 : 55.6	histosol	adventurers 1 1024a	13.58 (1.81 ; 24.62)	6.7	0.82	0.44	200	0.49	NA	0	vegetables	85; 20	3.518	5.651	-5.791
61	4	52.5302:-0.1822	15.0 : 26.2 : 58.7	histosol	adventurers 1 1024a	13.60 (1.60 ; 21.75)	7.1	0.71	0.39	420	0.61	NA	0	vegetables	70; 25	1.868	3.446	-0.799
62	4	52.5288:-0.1868	19.5 : 35.1 : 45.4	histosol	adventurers 1 1024a	12.84 (1.47 ; 18.85)	7.2	0.82	0.33	129	0.61	NA	0	arable	10; 20	0.927	-0.708	0.358
63	4	52.2569:-0.7382	34.4 : 28.1 : 37.6	luvisol	ashley 572q	9.69 (0.40 ; 3.88)	5.8	0.56	0.10	481	0.90	NA	0	forestry	60; 25	2.793	0.534	-0.672
66	4	50.6674:-3.3575	51.0 : 22.1 : 26.9	podzol	goldstone 631e	18.20 (0.46 ; 8.39)	4.6	1.12	0.18	557	0.90	NA	0	forestry	60; 30	1.999	1.519	-1.529
67	4	51.3809:-0.8360	88.3 : 9.9 : 1.7	podzol	holidays hill 643a	27.74 (0.28 ; 7.66)	4.6	0.78	0.15	373	0.90	NA	0	forestry	80; 45	0.759	1.314	-1.983
