# Supplementary material for: Distinct respiratory responses of soils to complex organic substrate are governed predominantly by soil architecture and its microbial community
Source: Soil Biol Biochem. 2016 Dec;103:493–501. doi: 10.1016/j.soilbio.2016.09.015 (PMC5113515; doi:10.1016/j.soilbio.2016.09.015)
Supplement: Table S2 — Model parameters and associated uncertainty measures for each sample site. [file mmc2.pdf]

SoilNo	k	k variance	B	B variance	τ_2	τ_2 variance	τ_3	$\tau 3$ variance	A_2	A_2 variance	A_3	A_3 variance	$Area_2$	$Area_3$
1	0.393	0.00132	6.41	0.079	7.12	1.018	49.26	0.178	0.38	0.0116	2.61	0.0006	2.70	204.96
2	0.349	0.00974	3.38	0.106	6.08	0.312	37.11	0.116	0.83	0.0468	3.06	0.0007	5.61	200.77
3	0.443	0.10103	4.23	0.329	5.32	3.631	48.00	0.367	1.21	0.4217	2.26	0.0006	11.43	203.88
4	0.691	0.02730	13.36	0.256	4.67	4.142	39.48	0.202	1.49	0.1813	2.37	0.0008	15.02	120.91
5	0.292	0.00612	3.34	0.069	9.11	0.406	30.85	0.745	1.36	0.0235	1.30	0.0020	16.89	34.53
6	0.300	0.00517	2.66	0.058	8.84	0.313	31.22	4.220	1.03	0.0245	0.97	0.0010	10.60	50.50
7	0.526	0.33716	5.75	0.356	4.93	29.173	57.83	0.709	1.18	1.5258	1.94	0.0006	12.55	199.72
8	0.445	0.08090	4.60	0.155	7.05	29.185	65.04	0.829	0.78	0.1717	1.99	0.0007	11.95	180.21
9	0.296	0.00395	4.38	0.078	8.23	0.237	35.70	0.328	1.63	0.0379	2.40	0.0008	17.85	117.89
10	0.201	0.00461	3.64	0.063	6.11	0.011	28.20	0.102	2.97	0.1217	3.92	0.0020	19.21	133.59
11	0.100	0.00043	2.92	0.016	5.36	0.015	28.63	0.562	1.98	0.0257	2.03	0.0104	10.35	61.31
12	0.253	0.02854	2.56	0.116	5.28	0.012	26.83	0.026	3.35	0.2455	6.34	0.0017	20.48	195.81
13	0.173	0.00220	2.46	0.039	9.43	0.076	32.76	0.116	1.48	0.0307	2.57	0.0012	13.32	95.03
14	0.215	0.00287	3.20	0.056	6.92	0.017	27.67	0.050	2.31	0.0412	4.14	0.0013	14.11	148.08
15	0.395	0.00468	3.86	0.077	7.43	0.048	32.01	0.147	2.35	0.0116	1.81	0.0011	20.51	61.22
16	0.175	0.00353	1.80	0.037	8.34	0.011	30.01	0.068	2.77	0.0247	3.63	0.0014	18.33	133.20
17	0.352	0.00244	6.97	0.094	7.41	0.185	34.37	0.114	1.88	0.0311	2.53	0.0010	18.99	96.56
18	0.409	0.00475	4.03	0.088	7.35	0.115	33.28	0.302	1.49	0.0119	1.63	0.0008	12.69	71.46
19	0.265	0.00357	2.99	0.061	7.62	0.047	32.43	0.048	1.77	0.0261	3.66	0.0011	12.57	150.57
20	0.385	0.00091	9.22	0.078	7.16	0.163	32.62	0.077	1.32	0.0156	2.67	0.0010	11.16	100.56
21	0.731	0.01113	10.54	0.224	5.90	0.295	37.99	0.208	2.57	0.0120	2.59	0.0009	30.60	115.32
22	0.302	0.00348	5.67	0.096	6.56	0.128	28.36	0.061	1.65	0.0729	3.67	0.0010	13.19	134.88
23	0.308	0.00592	2.49	0.060	8.09	0.050	33.29	0.059	2.06	0.0151	2.43	0.0012	17.72	76.22
24	0.602	0.17149	2.38	0.254	5.06	15.296	29.89	0.713	0.28	0.0639	1.97	0.0007	2.29	116.37
25	0.602	0.04462	3.08	0.123	8.92	1.164	45.48	18.313	1.07	0.0064	0.94	0.0032	20.15	57.55
26	0.486	0.04455	6.08	0.385	4.32	1.362	39.46	0.107	1.03	0.3866	3.06	0.0008	6.91	202.92
27	0.269	0.00497	4.19	0.089	7.63	0.949	56.58	0.720	0.77	0.0700	2.11	0.0007	7.56	215.84
28	0.263	0.00340	3.24	0.069	8.45	1.330	49.51	0.390	0.43	0.0255	2.50	0.0008	3.93	205.28
29	0.364	0.02420	1.81	0.088	9.41	0.614	28.97	31.524	1.01	0.0088	0.26	0.0040	12.46	6.30
30	0.146	0.01113	0.69	0.026	12.54	521.120	20.00	5753.035	0.25	5.4382	0.17	3.0609	2.65	2.91
31	0.293	0.00336	4.35	0.076	6.89	0.069	31.34	0.079	1.75	0.0412	2.34	0.0010	13.25	83.26
32	0.145	0.00622	0.50	0.018	18.20	3.594	49.03	0.351	0.26	0.0068	2.50	0.0007	3.79	139.26
33	0.822	0.06618	2.29	0.282	7.73	0.782	32.53	0.192	0.55	0.0056	3.48	0.0011	5.77	157.20
34	0.586	0.01462	11.16	0.303	4.76	1.228	37.76	0.197	1.48	0.1455	2.18	0.0007	12.31	122.47
35	0.307	0.00521	3.43	0.080	6.73	0.188	36.70	0.068	0.95	0.0351	3.75	0.0009	6.65	224.06
36	0.394	0.00237	6.31	0.084	7.49	0.141	33.64	0.146	1.74	0.0143	1.91	0.0011	16.85	66.06
37	0.264	0.00247	3.96	0.059	8.07	0.062	29.25	0.122	2.04	0.0293	2.38	0.0012	18.20	74.56
38	0.580	0.03466	6.48	0.253	5.91	1.803	34.72	2.204	1.46	0.0575	1.02	0.0011	16.20	45.70
39	0.206	0.00806	2.13	0.057	8.92	2.057	31.60	6.204	0.45	0.0683	1.03	0.0010	4.79	62.67
40	0.642	0.03547	7.27	0.271	5.34	1.022	36.15	0.155	1.77	0.0619	3.26	0.0012	17.84	162.56
41	0.339	0.00809	5.58	0.129	7.18	4.819	33.30	20.818	0.60	0.1249	0.69	0.0010	6.68	49.79
42	0.375	0.00275	6.01	0.092	7.10	0.260	33.59	0.264	1.21	0.0239	2.10	0.0008	10.76	103.32
43	0.374	0.00338	5.45	0.088	6.95	0.178	33.57	0.281	1.44	0.0259	2.01	0.0008	12.54	99.98
44	0.781	0.19189	4.88	0.275	4.55	5.879	32.09	13.914	0.96	0.1100	0.60	0.0010	9.43	35.14
45	0.224	0.00193	4.60	0.062	7.56	0.118	30.43	0.091	1.31	0.0550	3.39	0.0010	10.57	141.72
46	0.177	0.00590	1.61	0.045	7.43	0.008	27.83	0.094	3.02	0.0316	3.63	0.0021	16.65	123.85
47	0.168	0.00209	3.66	0.049	7.82	0.042	30.82	0.228	1.65	0.0817	1.83	0.0022	13.14	54.16
48	0.100	0.00823	1.17	0.034	7.53	0.005	25.78	0.925	3.64	0.0409	2.32	0.0426	21.43	76.10
49	0.317	0.00189	4.51	0.063	8.01	0.055	32.01	0.125	1.93	0.0145	1.93	0.0011	16.33	64.85
50	0.296	0.00565	2.45	0.054	9.93	0.670	28.27	1019.368	0.85	0.0631	0.16	0.0056	10.61	9.79
51	0.145	0.00404	1.09	0.025	10.57	0.725	20.00	371.727	0.95	0.0955	0.11	0.0171	7.42	1.93
52	0.706	0.02143	14.23	0.266	4.71	4.211	42.70	0.533	1.35	0.1402	1.66	0.0007	13.71	104.52
53	0.528	0.01656	10.21	0.177	7.27	10.282	38.90	0.709	0.98	0.0486	3.26	0.0037	15.36	141.00
54	0.577	0.01366	2.82	0.100	10.47	0.757	36.57	970.233	0.79	0.1351	0.30	0.0091	13.45	23.35
55	0.201	0.00117	4.70	0.049	8.09	0.427	35.19	0.097	0.62	0.0444	2.86	0.0009	5.04	130.07
56	0.427	0.00132	10.21	0.110	6.84	0.227	38.44	0.181	1.36	0.0191	2.56	0.0009	12.09	144.16
57	0.267	0.00991	3.30	0.090	6.86	0.190	31.31	0.061	1.48	0.1017	3.45	0.0012	12.80	129.33
58	0.491	0.02155	3.84	0.148	6.06	0.330	31.69	0.155	1.48	0.0290	2.19	0.0009	13.01	84.81
59	0.381	0.00416	4.57	0.091	6.61	0.143	31.73	0.133	1.26	0.0229	2.40	0.0008	9.35	108.95
60	0.343	0.00477	1.85	0.043	11.78	0.176	36.72	1.331	1.17	0.0060	1.29	0.0009	14.05	58.39
61	0.934	0.02346	3.11	0.164	19.66	1971.874	70.00	8309629.135	0.30	37.5946	0.08	2.4976	12.52	12.70
62	0.851	0.02177	3.40	0.146	12.90	20.370	51.69	47975.224	0.59	1.0283	0.15	0.0595	15.83	16.77
63	0.196	0.02782	1.90	0.087	11.22	26.097	70.00	10507.588	0.44	0.2219	0.05	0.0007	8.06	7.39
64	0.299	0.00120	6.87	0.069	7.92	0.084	32.06	0.084	2.08	0.0275	3.30	0.0010	19.81	125.10
65	0.637	0.01667	6.92	0.255	5.67	0.181	32.87	0.082	2.53	0.0202	2.53	0.0011	23.81	86.31
66	0.193	0.01117	4.52	0.064	20.00	3840.361	43.20	351462.292	0.79	953.6783	1.20	399.2758	31.82	100.24
67	0.247	0.02419	2.42	0.090	20.00	22.277	32.67	67280.530	0.63	310.3778	0.94	163.6019	18.95	50.67
