# Supplementary material for: Distinct respiratory responses of soils to complex organic substrate are governed predominantly by soil architecture and its microbial community
Source: Soil Biol Biochem. 2016 Dec;103:493–501. doi: 10.1016/j.soilbio.2016.09.015 (PMC5113515; doi:10.1016/j.soilbio.2016.09.015)
Supplement: Table S3 — Loadings extracted from the phenotypic principal components analysis. [file mmc3.pdf]

Fatty acid	Phenotypic PC1	Phenotypic PC2	Phenotypic PC3
C14:0	0.266	-0.352	0.680
C15:0i	-0.499	-0.305	0.501
C15:0ai	-0.557	-0.133	0.388
C15:0	0.656	-0.195	0.526
C16:1	0.509	-0.087	0.648
C16:1ω11c	0.210	0.056	0.244
C16:0i	-0.074	-0.078	0.183
C16:1ω11t	-0.152	0.180	0.138
C16:1ω7c	-0.285	0.017	0.250
C16:1ω5	-0.333	0.156	0.294
C16:0i 2	0.095	-0.482	-0.590
C16:0(10me)	-0.406	0.614	0.263
Cpthalate	-0.460	0.350	0.279
C17:0i	0.564	0.442	-0.057
C17:0ai	0.026	0.750	0.384
C17:0brb	-0.111	0.796	-0.084
C17:1ω8c	0.567	0.641	-0.097
C17:0cy	-0.511	0.572	-0.086
C17:1ω8t	0.558	0.008	0.569
C17:0	0.619	0.264	0.118
C17:0(12me)	0.291	0.519	0.088
C17:0(10me)	0.442	0.462	-0.042
C18:3(5,10,12)	0.633	-0.285	-0.596
C18:2(6,9)	0.273	-0.131	-0.441
C18:1ω6	-0.738	0.029	-0.143
C18:1ω9	-0.807	0.152	0.050
C18:1ω13	0.040	0.347	-0.338
C18:0	0.229	0.374	-0.287
C19:1ω6	0.497	0.117	-0.065
C18:0(10me)	0.063	0.680	-0.181
C19:0cy	-0.341	-0.191	0.066
C20:1ω9	0.201	-0.238	-0.209
C20:1ω9 2	0.461	-0.329	0.322
C20:0	0.627	0.130	0.243
