# Supplementary figures and images for: Distinct respiratory responses of soils to complex organic substrate are governed predominantly by soil architecture and its microbial community
Source: Soil Biol Biochem. 2016 Dec;103:493–501. doi: 10.1016/j.soilbio.2016.09.015 (PMC5113515; doi:10.1016/j.soilbio.2016.09.015)

## Slide 1
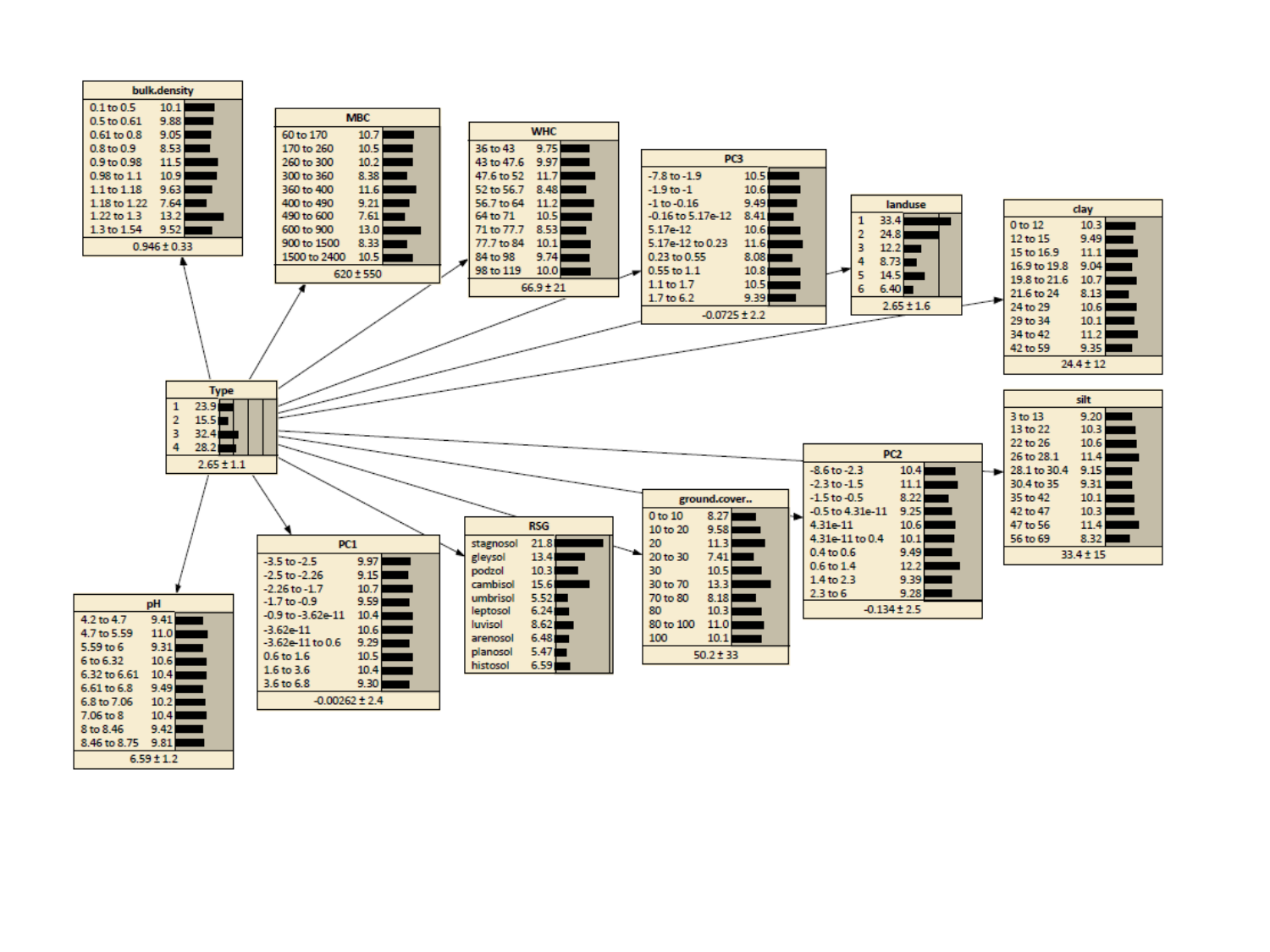

Supplement: Fig. S1 — Final parsimonious Bayesian belief network diagram; the arrows show the structure of the model with all 12 predictor variables being directly related to respiratory Type. The bars show the distribution of predictor variables across all 67 sites, the range of each parameter value is divided into 10 bins the limits of which are shown to the left of the left hand column with the percentage of sites falling in each bin shown to the right of the left hand column. MBC – microbial biomass carbon, WHC – water holding capacity, RSG – representative soil group, in land use node numbers relate to categories of land use as listed in Table 2; 1 – pasture, 2 – other, 3 – arable, 4 – moorland, 5 – forestry, and 6 – vegetables. [file mmc4.pptx]
